# Estimation of Lead Exposure Intensity by Industry Using Nationwide Exposure Databases in Korea

**DOI:** 10.1016/j.shaw.2021.07.008

**Published:** 2021-07-17

**Authors:** Dong-Hee Koh, Ju-Hyun Park, Sang-Gil Lee, Hwan-Cheol Kim, Hyejung Jung, Inah Kim, Sangjun Choi, Donguk Park

**Affiliations:** 1 Department of Occupational and Environmental Medicine, International St. Mary’s Hospital, Catholic Kwandong University, Incheon, Republic of Korea; 2 Department of Statistics, Dongguk University, Seoul, Republic of Korea; 3 Occupational Safety and Health Research Institute, Korea Occupational Safety and Health Agency, Ulsan, Republic of Korea; 4 Department of Occupational and Environmental Medicine, Inha University, Incheon, Republic of Korea; 5 Department of Occupational and Environmental Medicine, College of Medicine, Hanyang University, Seoul, Republic of Korea; 6 Department of Preventive Medicine, College of Medicine, The Catholic University of Korea, Seoul, Republic of Korea; 7 Department of Environmental Health, Korea National Open University, Seoul, Republic of Korea

**Keywords:** Cancer, Carcinogen, Exposure, Occupational cancer, Occupational exposure

## Abstract

**Background:**

In a previous study, we estimated exposure prevalence and the number of workers exposed to carcinogens by industry in Korea. The present study aimed to evaluate the optimal exposure intensity indicators of airborne lead exposure by comparing to blood lead measurements for the future development of the carcinogen exposure intensity database.

**Methods:**

Data concerning airborne lead measurements and blood lead levels were collected from nationwide occupational exposure databases, compiled between 2015 and 2016. Summary statistics, including the arithmetic mean (AM), geometric mean (GM), and 95th percentile level (X95) were calculated by industry both for airborne lead and blood lead measurements. Since many measurements were below the limits of detection (LODs), the simple replacement with half of the LOD and maximum likelihood estimation (MLE) methods were used for statistical analysis. For examining the optimal exposure indicator of airborne lead exposure, blood lead levels were used as reference data for subsequent rank correlation analyses.

**Results:**

A total of 19,637 airborne lead measurements and 32,848 blood lead measurements were used. In general, simple replacement showed a higher correlation than MLE. The results showed that AM and X95 using simple replacement could be used as optimal exposure intensity indicators, while X95 showed better correlations than AM in industries with 20 or more measurements.

**Conclusion:**

Our results showed that AM or X95 could be potential candidates for exposure intensity indicators in the Korean carcinogen exposure database. Especially, X95 is an optimal indicator where there are enough measurements to compute X95 values.

## 1 Introduction

Cancers are among the leading causes of death worldwide and can lead to a heavy economic burden on patients and society [1]. Cancer is a multifactorial disease caused by genetic and environmental factors [2]. Among these factors, occupational exposure is estimated to contribute to 2–8% of all cancers [[3], [4], [5]]. Especially, occupational exposure is estimated to account for 14.5% of lung cancer cases and 32.7% of sinonasal cancer cases [5]. The critical fact is that occupational cancers are largely avoidable.

In an effort to prevent occupational cancers, many countries have developed occupational carcinogen exposure surveillance systems, such as the Finnish Job-Exposure Matrix and CARcinogen EXposure (CAREX) [[6], [7], [8]]. Furthermore, the Korean CAREX (K-CAREX) was developed for 20 definite human carcinogens (group 1) for Korean working populations [9]; however, it focused on the exposure prevalence and the number of exposed workers, while the exposure intensity had not been established.

In the present study, we aimed to examine various exposure intensity indicators to be used in the K-CAREX. To do so, we estimated exposure intensity by industry using lead as an exemplary carcinogen. We selected lead because there were relatively abundant measurements both for airborne and blood lead. Also, blood lead is known to reflect airborne lead exposure well [10]. Lead is classified as a probable carcinogen (Group 2A) related to excess risk of stomach, kidney, and brain cancers [11]. The results can be applied to other carcinogens for the future development of the K-CAREX exposure intensity.

## 2 Methods

### 2.1 Data sources

There is an occupational exposure surveillance system administered by the government in Korea. Every workplace should undergo periodic workplace exposure monitoring for 190 chemical agents and two physical agents. Workplaces are requested to undergo environmental monitoring basically twice a year, and the schedules change according to the hazardous agent and previous compliance/violation conditions [12]. The measurement data are centrally collected by the Korea Occupational Safety and Health Agency (KOSHA) as an electronic database since 2002, which is referred to as the Work Environment Measurement Database (WEMD) [13]. The WEMD contains information such as industry, work process, sampling site, sampling time, and time-weighted average. We extracted airborne lead measurement data, including sampling time, industrial code, and concentration, from the WEMD between 2015 and 2016.

There is an occupational health surveillance system administered by the government in Korea. Every worker exposed to any one of 176 physical and chemical agents (including night-shift work) should undergo a periodic health examination for the agent(s). The results are centrally collected by the KOSHA and deposited into an electronic database since 2000, which is named the Special Health Examination Database (SHED) [14]. The SHED contains information such as blood tests and biological monitoring results. We extracted blood lead level data from the SHED between 2015 and 2016.

### 2.2 Industrial classification

In the WEMD and the SHED between 2015 and 2016, data were recorded using the Korean Standard Industrial Classification (KSIC-9), which is almost identical to the International Standard Industrial Classification (ISIC, 4th revision) because KSIC-9 was developed based on the ISIC. We used a 3-digit ISIC industrial group as a standard industrial classification (SIC) for subsequent analyses.

### 2.3 Data cleaning and treatment

In workplace monitoring in Korea, a personal sample is typically required to be measured for at least 6 hours. We excluded measurements taken for <4 hours or >12 hours. We also excluded measurements with incorrect SIC codes.

We restricted analyses to workplaces having data pertaining to both airborne and blood lead. Then, industries with at least two nonmissing, noncensored, distinct levels were included for analyses.

Each workplace monitoring occurrence might have its level of a limit of detection (LOD); however, that information is not available in the WEMD and SHED. Therefore, for airborne lead, we assumed a single LOD according to a standard sampling and analytical method of the KOSHA (KOSHA GUIDE A-2-2012), which is largely based on the US National Institute of Occupational Safety and Health (NIOSH) method [15]. The LOD is estimated assuming 6 hours sampling time using a mixed cellulose filter, resulting in 1.8 μg/m^3^.

For blood lead level (BLL), similar to airborne lead measurements, a single LOD was assumed. For measuring BLL, a standard analytical method of the KOSHA (KOSHA GUIDE H-21-2011) was employed, which is largely based on the US NIOSH method using graphite furnace atomic absorption spectrophotometry [16]. The LOD of BLL was estimated at 0.85 μg/dL.

### 2.4 Statistical analysis

Environmental measurements are known to be log-normally distributed [17]. Since the lead measurement data were left-censored below the LOD, we examined a log-normality of overall data by comparing an empirical cumulative distribution function (CDF) with a hypothesized fitted CDF using the “EnvStats” package [17] of R [18]. Also, we examined the ordered data (the empirical quantiles) vs. the corresponding quantiles from the theoretical probability distribution using a quantile-quantile plot (Q-Q plot).

We calculated summary statistics for each industry, including arithmetic mean (AM), standard deviation (SD), geometric mean (GM), geometric standard deviation (GSD), and 95th percentile level (X95). Korean occupational exposure limit (OEL) of lead was 50 μg/m^3^. The biological exposure index (BEI) of BLL was 30 μg/dL in line with the BEI of the American Conference of Governmental Industrial Hygienists (ACGIH), although the ACGIH BEI has been reduced from 30 μg/dL to 20 μg/dL since 2016 [19].

Summary statistics were calculated using two methods: simple replacement and the maximum likelihood estimation (MLE). Levels below the LOD were replaced with the value of LOD/2 for a simple replacement method [20]. For the MLE method, levels below the LOD were treated as censored, then used for estimating distribution parameters [17,21]. For MLE, GM and GSD were computed using a function of “elnormCensored” of “EnvStats” package of R. Estimated X95 was computed using a function of “eqlnormCensored” of “EnvStats” package. In cases where the industry had no censored value, “elnorm”, and “eqlnorm” functions were used instead.

Spearman rank correlation analyses were performed across industries to compare rank orders of the summary statistics (AM, GM, and X95) of airborne lead with those of blood lead, which were treated as the reference. Furthermore, these analyses were repeated, restricting for industries with ≥20 and ≥100 measurements, respectively.

## 3 Results

The overall number of measurements for airborne lead and blood lead were 47,575 and 56,121, respectively. Finally, excluding measurements not eligible for analysis, a total of 19,637 airborne lead (41%) and 32,848 blood lead measurements (59%) were used for analysis. Censoring rates of airborne lead and blood lead measurements were 78.0% and 13.5%, respectively.

**Fig. 1:**
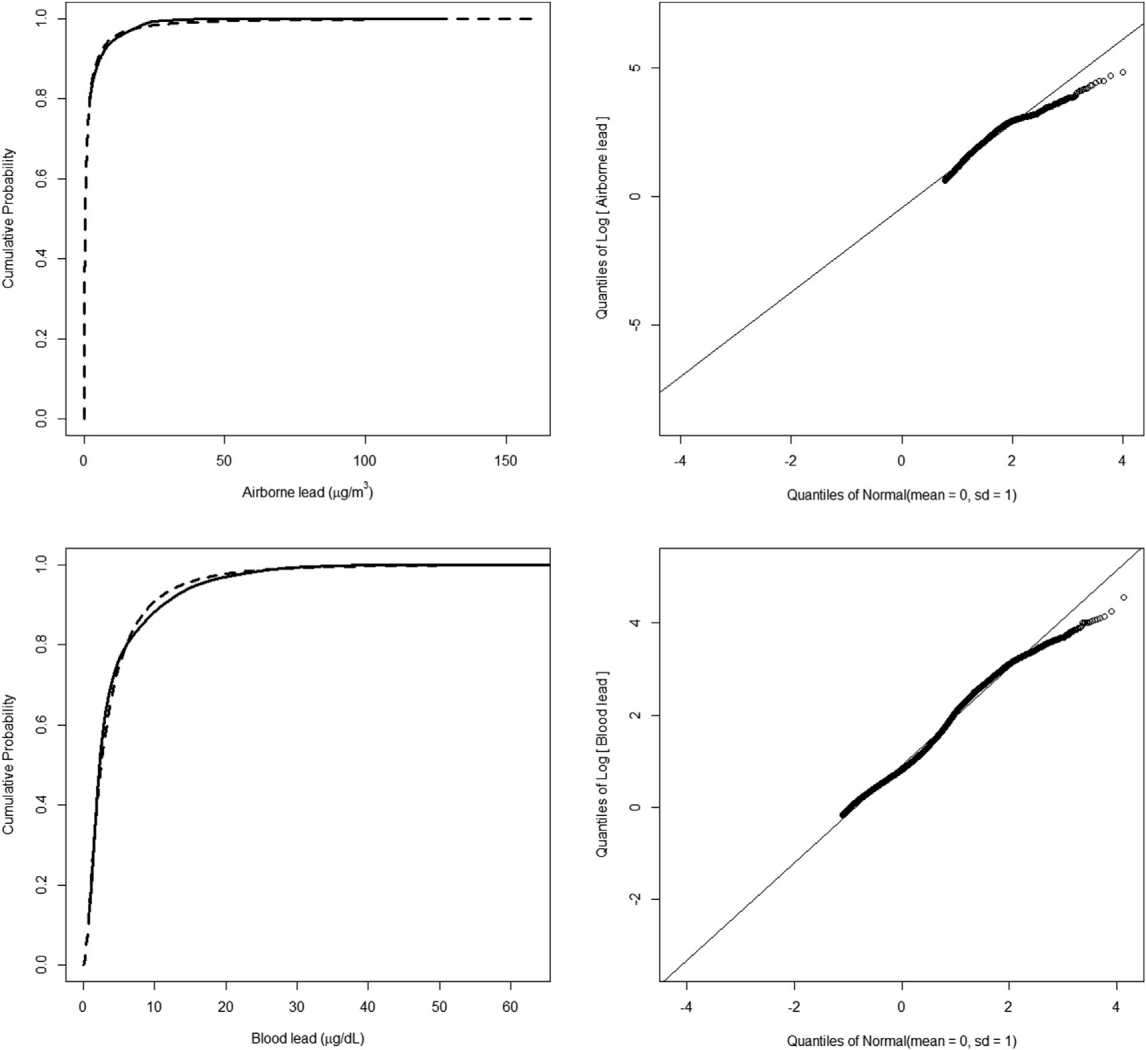
Empirical CDF for lead (censored: solid line) with fitted log-normal CDF (dash line) (Left). Normal Q-Q plot for left-censored log-transformed lead measurements (Right).

The CDF and the Q-Q plot of overall measurements show that lead data have a distribution that is close to a log-normal one (Fig. 1).

Table 1 shows the results of Spearman rank correlation between airborne lead and blood lead measurements. In general, simple replacement showed a better correlation than MLE. In terms of exposure intensity indicator, AM showed the highest correlation with all data, while X95 showed the highest correlation when restricting the analysis to industries with 20 or more measurements. The correlation coefficients further increased when restricting the analysis to industries with 100 or more measurements, although the number of industries eligible for the analysis decreased. In general, AM with simple replacement or X95 with simple replacement can be candidates for exposure intensity indicators in the WEMD database. However, considering that a small number of measurements render the intensity estimates unstable, it is reasonable to restrict analyses to industries with 20 or more measurements and employ X95 as an optimal exposure intensity indicator.

**Table 1:** Results of Spearman rank correlation between airborne lead and blood lead measurements across industries

	All industries (64 industries, 19,637 airborne and 32,848 blood lead measurements)				Industries with ≥20 measurements (49 industries, 19,329 airborne and 32,560 blood lead measurements)				Industries with ≥100 measurements (26 industries, 17,283 airborne and 28,793 blood lead measurements)			
	Simple replacement		MLE		Simple replacement		MLE		Simple replacement		MLE	
	r	*p*	r	*p*	r	*p*	r	*p*	r	*p*	r	*p*
AM	0.470	<0.001			0.553	<0.001			0.653	<0.001		
GM	0.426	<0.001	0.227	0.071	0.500	<0.001	0.194	0.181	0.635	0.001	0.411	0.038
X95	0.443	<0.001	0.448	<0.001	0.581	<0.001	0.578	<0.001	0.722	<0.001	0.649	<0.001

AM, arithmetic mean; GM, geometric mean; MLE, maximum likelihood estimation; r, Spearman rank correlation coefficient; X95, 95th percentile level.

Summary statistics of measurements of the top-ten upper and lower industries according to X95 with the simple replacement for airborne lead are presented in Table 2. The “Cast of Metals” industrial group (SIC 243) showed the highest X95 level of 35.54 μg/m^3^. In general, industries involving high exposures showed low censoring rates, as well as high AM and GM values. Detailed results of airborne and blood lead by the 3-digit industrial group are available online in Supplemental Table 1.

**Table 2:** Top-ten high- and low-exposure industries based on X95 of airborne lead using simple replacement methods (industry with ≥20 measurements)

		Airborne lead											Blood lead										
		Censoring rate			Simple replacement					Maximum likelihood estimation			Censoring rate			Simple replacement					Maximum likelihood estimation		
SIC	Explanation	Censored	Total	Rate (%)	AM	SD	GM	GSD	X95	GM	GSD	X95	Censored	Total	Rate (%)	AM	SD	GM	GSD	X95	GM	GSD	X95
***High-exposure industry***																							
243	Cast of Metals	192	353	54.39	6.60	11.04	2.49	3.66	32.54	1.55	6.62	34.72	5	206	2.43	8.20	9.00	4.91	2.78	26.02	4.95	2.73	25.84
251	Manufacture of Structural Metal Products, Tanks, Reservoirs and Steam Generators	119	741	16.06	11.68	8.58	7.57	3.05	23.10	7.82	2.91	45.22	0	63	0.00	6.04	4.98	4.89	1.87	16.29	4.89	1.87	13.67
282	Manufacture of Primary Cells and Batteries and Accumulators	304	734	41.42	6.11	8.14	3.08	3.24	22.94	2.75	3.99	26.77	12	2842	0.42	10.65	5.91	8.96	1.90	22.35	8.98	1.88	25.41
242	Manufacture of Basic Precious and Nonferrous Metals	380	821	46.29	5.97	8.88	2.79	3.33	21.85	2.28	4.55	27.63	42	3472	1.21	10.70	9.02	7.44	2.48	28.40	7.48	2.44	32.50
201	Manufacture of Basic Chemicals	77	145	53.10	4.40	5.13	2.34	3.04	16.88	1.80	4.43	20.82	5	271	1.85	6.94	7.99	3.97	2.86	23.86	3.99	2.84	22.20
222	Manufacture of Plastic Products	355	485	73.20	3.17	5.91	1.54	2.66	14.62	0.55	7.19	14.20	3	358	0.84	7.66	7.22	5.18	2.46	22.59	5.19	2.44	22.53
239	Manufacture of Other Nonmetallic Mineral Products	34	49	69.39	5.55	18.26	1.71	3.14	13.59	0.61	8.58	21.08	19	743	2.56	7.22	4.72	5.69	2.15	16.28	5.76	2.07	19.13
241	Manufacture of Basic Iron and Steel	611	990	61.72	3.33	4.80	1.81	2.69	13.06	1.21	4.44	14.02	1365	6965	19.60	2.01	2.57	1.42	2.20	5.01	1.50	2.06	4.94
729	Other Scientific and Technical Services	32	38	84.21	2.41	4.05	1.29	2.42	13.02	0.13	14.29	10.54	32	161	19.88	1.81	1.11	1.45	2.05	3.93	1.56	1.82	4.21
259	Manufacture of Other Metal Products; Metal Working Service Activities	414	577	71.75	2.61	4.14	1.48	2.42	11.56	0.73	5.08	10.50	81	870	9.31	4.38	4.27	3.11	2.41	10.45	3.21	2.26	12.27
***Low-exposure industry***																							
491	Interurban Rail Transportation	139	160	86.88	1.18	0.91	1.04	1.51	2.61	0.67	2.42	2.86	3	43	6.98	1.60	0.58	1.47	1.57	2.52	1.53	1.42	2.71
261	Manufacture of Semiconductor	331	357	92.72	1.17	1.43	1.00	1.50	2.60	0.17	5.14	2.47	1176	1988	59.15	1.02	0.97	0.75	2.09	2.90	0.71	2.44	3.08
265	Manufacture of Electronic Video and Audio Equipment	140	152	92.11	1.25	1.98	1.01	1.55	2.54	0.16	5.55	2.68	2	150	1.33	2.51	1.29	2.23	1.65	4.41	2.24	1.61	4.91
211	Manufacture of Medicinal Chemicals, Antibiotics and Biological Products	85	92	92.39	1.10	0.76	0.99	1.43	2.49	0.30	3.49	2.37	9	197	4.57	2.34	1.31	2.02	1.75	4.94	2.07	1.66	4.76
381	Waste Collection	61	65	93.85	1.26	2.28	0.99	1.55	1.64	0.07	7.81	2.20	0	38	0.00	4.89	1.51	4.63	1.41	7.10	4.63	1.41	8.18
582	Software Development and Supply	47	50	94.00	1.49	2.81	1.02	1.76	1.45	0.01	23.94	2.43	0	58	0.00	2.47	0.84	2.33	1.42	4.33	2.33	1.42	4.15
313	Manufacture of Aircraft, Spacecraft and its Parts	72	76	94.74	0.99	0.40	0.95	1.27	1.18	0.54	2.12	1.84	63	685	9.20	1.89	1.01	1.63	1.78	3.74	1.71	1.63	3.81
212	Manufacture of Medicaments	84	88	95.45	1.01	0.54	0.95	1.32	0.90	0.19	3.81	1.71	182	309	58.90	0.96	0.81	0.73	2.01	2.67	0.72	2.23	2.71
721	Architectural, Engineering and Related Technical Services	52	54	96.30	0.98	0.43	0.94	1.25	0.90	0.27	2.91	1.55	18	46	39.13	1.14	0.78	0.91	1.97	2.86	1.02	1.78	2.65
861	Hospital Activities	281	291	96.56	0.96	0.36	0.93	1.22	0.90	0.36	2.41	1.54	95	396	23.99	1.92	1.36	1.45	2.23	4.26	1.56	2.05	5.06

AM, arithmetic mean; GM, geometric mean; GSD, geometric standard deviation; SD, standard deviation; SIC, International Standard Industrial Classification (4th revision); X95, 95th percentile level.

## 4 Discussion

Using nationwide occupational exposure databases, in the present study, we estimated lead exposure intensity by industry. We examined AM, GM, and X95 as indicators of exposure intensity by comparing airborne lead to blood lead as a reference. Our approach is reasonable to determine an optimal exposure intensity indicator across industries, which will be used to develop exposure intensity to other carcinogens in the K-CAREX.

There is currently no single optimal estimation method that is used for the values less than the LOD, and the accuracy of any estimation is dependent on the censoring rate, sample size, and LOD type (for instance, single or multiple). However, for AM and the 95th percentile, it is reported that the MLE-based method is largely recommended regardless of censoring rate and sample size [21]. Furthermore, for estimating upper extreme quantiles based on a small sample of less than 30, reporting the MLE of quantiles under the log-normal assumption is suggested [22]. However, in the present study, simple replacement showed a better correlation with blood lead compared to MLE, which is contrary to the aforementioned reports, and we could not establish the cause of this discrepancy. In terms of statistical distribution, the MLE method is based on the log-normality assumption; however, in reality, the assumption did not hold in every industry, which might have led to inconsistent results. In terms of study design, previous studies had largely employed a simulation method to compare incomplete data with values less than the LOD and complete dataset. However, our study compared airborne measurements with biomonitoring data.

AM with simple replacement and X95 with simple replacement are selected as candidates as exposure intensity indicators in the Korean CAREX. Regarding AM, the percentage bias (%bias) is strongly dependent on the percentage of censored observations, which seems to be independent of sample size. When the percentage of a censored value is greater than 50%, a substantial negative bias with a %bias lower than −20% is reported. Regarding the 95th percentile, the %bias is lower than that of AM [23]. Therefore, considering that there is a high proportion of values below the LOD in the WEMD, we prefer X95 with a simple replacement as a default exposure intensity indicator.

It is suggested that monitoring initial exposure should consist of at least six to ten measurements taken from randomly chosen workers [24]. In general, 10–20 measurements per observational group (for instance, two measurements from 5–10 randomly selected persons) are regarded to be sufficient for an initial exposure assessment [25]. Although airborne samples were nonrandomly selected, we conclude that it is reasonable to assign X95 only to industries with 20 or more measurements. In fact, restricting to industries with 20 or more measurements seems to be intuitively appropriate because it requires at least 20 samples for calculating the 95th percentile value.

Strength of correlation increases when we restrict our analysis to industries that have more measurements, while information is lost by decreasing the number of assessed industries. There is a trade-off between the strength of correlation and the amount of information. We, therefore, prefer restricting to industries with 20 or more measurements because this increases the strength of correlation while not losing many industries.

Although the strength of the correlation seems fair [26], in some industries, the airborne lead level was not significantly correlated with BLL. For instance, “Manufacture of Basic Iron and Steel” showed a high rank of airborne X95 lead level but showed a low blood X95 lead level (Table 2). There would be several factors associated with the discrepancy, such as ventilation and respirator use. However, in this case, we suspect that health surveillance might be overly performed where only a small proportion of workers are exposed. This is represented by a high censoring rate of blood lead levels, which resulted in low X95 blood lead levels. Therefore, it is important to recognize that our exposure intensity indicators only applied to exposed workers in an industry, but not all workers in that industry.

The strength of our study lies primarily in using large measurement databases. We used nationwide blood lead measurement data as a reference to be compared with nationwide airborne lead data. Besides, we examined various exposure indicators such as AM, GM, and X95, which may be used for evaluating exposure in various ways.

Our study has several limitations. First, work environment monitoring is conducted as a compliance measurement. Industrial hygienists select workers to be measured according to their own judgment, which is based on maximum potential exposure rather than random sampling. Using judgment sampling to select workers can result in an inability to compute precision, causing bias in estimates that are computed from these measurements [17]. Judgment sampling based on maximum potential exposure may lead to potential overestimation; on the other hand, because the cost of work environmental monitoring is paid by businesses, there is an inherent potential underestimation of exposure. We could not figure out the net effects of these factors, but we suspect that there is an underestimation of exposure. To cope with the potential underestimation, we considered analyzing data that only includes measurements that are above the LOD; however, this may only highlight the high exposure levels and poses a potential risk of overestimation. Therefore, we included all measurements in our analyses, and the results should be interpreted with caution considering this aspect.

Second, while workers to be measured are selected based on maximum potential exposure in workplace exposure monitoring, health examinees are selected based on minimum exposure, which means all workers exposed to the agent(s) should undergo special health examination regardless of exposure level. Considering the different sampling strategies, we used the Spearman rank correlation test rather than the Pearson correlation test.

Third, work environment measurement agencies are required to be equipped to perform atomic absorption spectrometry (AAS) by the Occupational Safety and Health Act in Korea. Therefore, airborne lead samples were quantified mainly by the AAS, while a small proportion of samples were analyzed by inductively coupled plasma spectrometry (ICP). The LOD of ICP is far lower than that of AAS [27]; however, we could not obtain those LODs, and therefore applied a single LOD based on the AAS method.

Fourth, we analyzed measurements from workplaces conducted both airborne lead and blood lead monitoring. However, workplaces that conducted only airborne lead monitoring showed lower summary statistics than workplaces that conducted both airborne lead and blood lead monitoring (data not shown). It may suggest that workplaces with high exposure are more likely to conduct blood lead monitoring. Therefore, our results may have a potential limitation in applying to all workplaces.

Fifth, there are potential errors in the coding of industries in the database. For instance, “Spinning of Textiles and Processing of Threads and Yarns” (SIC: 131) showed a high level of exposure; however, when further investigating the work process and sampling sites, we found that the industry involved the manufacturing of steel wires (rolling/heat treatment/chamfer) and was incorrectly coded. Therefore, we re-assigned these measurements to “Manufacture of Basic Iron and Steel” (SIC: 241). However, we could not check every industrial code and, therefore, the possibility of unchecked coding errors should be considered, especially in industries with small sample sizes.

The WEMD consists of a large proportion of measurements below LODs, which leads to difficulty in assessing and ranking exposure intensity. In the present study, we computed summary statistics of airborne lead exposure and tested optimal intensity indicators of airborne lead exposure by comparing to blood lead data across industries. This result may aid in establishing the K-CAREX exposure intensity, which would assess exposures to other carcinogens.

## Ethics

The study protocol was reviewed and approved by the Institutional Review Board of the Catholic Kwandong University, International St. Mary’s Hospital, Incheon, Korea (IS17QIMI0035).

## Conflicts of interest

The authors declare that they have no conflict of interests.
